# *NBAS* Variants Are Associated with Quantitative and Qualitative NK and B Cell Deficiency

**DOI:** 10.1007/s10875-021-01110-7

**Published:** 2021-08-13

**Authors:** Dominic Lenz, Jens Pahl, Fabian Hauck, Seham Alameer, Meena Balasubramanian, Ivo Baric, Nikolas Boy, Joseph A. Church, Ellen Crushell, Anke Dick, Felix Distelmaier, Jidnyasa Gujar, Giuseppe Indolfi, Eberhard Lurz, Bianca Peters, Tobias Schwerd, Daniele Serranti, Stefan Kölker, Christoph Klein, Georg F. Hoffmann, Holger Prokisch, Johann Greil, Adelheid Cerwenka, Thomas Giese, Christian Staufner

**Affiliations:** 1 Division of Neuropediatrics and Pediatric Metabolic Medicine, Center for Pediatric and Adolescent Medicine, University Hospital Heidelberg, Im Neuenheimer Feld 430, 69120 Heidelberg, Germany; 2 Department of Immunobiochemistry, Mannheim Institute for Innate Immunoscience (MI3), Medizinische Fakultät Mannheim, Universität Heidelberg, Mannheim, Germany; 3 Department of Pediatrics, Dr. Von Hauner Children’s Hospital, University Hospital, Ludwig-Maximilians-Universität München (LMU), Munich, Germany; 4 German Centre for Infection Research (DZIF), Munich, Germany; 5 Munich Centre for Rare Diseases (M-ZSELMU), University Hospital, Ludwig-Maximilians-Universität München, Munich, Germany; 6 Pediatric Department, Ministry of National Guard Health Affairs, Jeddah, Saudi Arabia; 7 King Abdullah International Medical Research Center, Jeddah, Saudi Arabia; 8 King Saud Bin Abdulaziz University for Health Sciences, Jeddah, Saudi Arabia; 9 Sheffield Clinical Genetics Service, Sheffield Children’s NHS Foundation Trust, Sheffield, UK; 10 Department of Oncology & Metabolism, University of Sheffield, Sheffield, UK; 11 Department of Pediatrics, School of Medicine, University Hospital Center Zagreb and University of Zagreb, Zagreb, Croatia; 12 Department of Pediatrics, Keck School of Medicine of the University of Southern California, and Children’s Hospital Los Angeles, Los Angeles, CA USA; 13 National Centre for Inherited Metabolic Disorders, Children’s Health Ireland At Temple Street and Crumlin, Dublin, Ireland; 14 Department of Pediatrics, University Hospital Würzburg, Würzburg, Germany; 15 Department of General Pediatrics, Neonatology and Pediatric Cardiology, University Children’s Hospital, Heinrich-Heine-University Düsseldorf, Düsseldorf, Germany; 16 Paediatric and Liver Unit, Meyer Children’s University Hospital of Florence, Firenze, Italy; 17 Institute of Human Genetics, Klinikum Rechts Der Isar, Technical University of Munich, Munich, Germany; 18 Institute of Human Genetics, Helmholtz Zentrum Munich, Neuherberg, Germany; 19 Department of Pediatric Hematology and Oncology, University Children’s Hospital, Heidelberg, Germany; 20 Institute of Immunology and German Center for Infection Research (DZIF), Heidelberg University Hospital, Heidelberg, Germany

**Keywords:** *NBAS*, inborn error of immunity, NK cell deficiency, B cell deficiency, vesicle trafficking, familial hemophagocytic lymphohistiocytosis

## Abstract

**Purpose:**

Biallelic pathogenic *NBAS* variants manifest as a multisystem disorder with heterogeneous clinical phenotypes such as recurrent acute liver failure, growth retardation, and susceptibility to infections. This study explores how NBAS-associated disease affects cells of the innate and adaptive immune system.

**Methods:**

Clinical and laboratory parameters were combined with functional multi-parametric immunophenotyping methods in fifteen NBAS-deficient patients to discover possible alterations in their immune system.

**Results:**

Our study revealed reduced absolute numbers of mature CD56^dim^ natural killer (NK) cells. Notably, the residual NK cell population in NBAS-deficient patients exerted a lower potential for activation and degranulation in response to K562 target cells, suggesting an NK cell–intrinsic role for NBAS in the release of cytotoxic granules. NBAS-deficient NK cell activation and degranulation was normalized upon pre-activation by IL-2 in vitro, suggesting that functional impairment was reversible. In addition, we observed a reduced number of naïve B cells in the peripheral blood associated with hypogammaglobulinemia.

**Conclusion:**

In summary, we demonstrate that pathogenic biallelic variants in *NBAS* are associated with dysfunctional NK cells as well as impaired adaptive humoral immunity.

**Supplementary Information:**

The online version contains supplementary material available at 10.1007/s10875-021-01110-7.

## Introduction

NBAS-associated disease is a rare autosomal recessive disorder with a broad spectrum of clinical symptoms, mainly involving liver, growth, skeletal system, nervous system, integument, immune system, and musculature. Biallelic pathogenic *NBAS* variants were first described in an isolated Russian population in 2010 causing a syndrome comprising short stature, optic atrophy, and Pelger-Huët anomaly (SOPH syndrome; MIM 614800) [1]. In 2015, *NBAS* variants were linked to fever-related recurrent acute liver failure [infantile liver failure syndrome 2 (ILFS2), MIM 616483] [2]. To date, NBAS-associated disease has been documented in at least 110 patients from 97 families and *NBAS* variants can be classified into three subgroups according to the affected part of the protein: “Sec39,” with a predominant hepatic phenotype (ILFS2), “C-terminal” with the multisystemic SOPH phenotype, and “β-propeller” with a combined phenotype (ILFS2-SOPH) [3].

Immunological symptoms and laboratory alterations such as frequent infections, hypogammaglobulinemia, low natural killer (NK) cell numbers, and neutropenia have been observed in more than 60% of patients with NBAS-associated disease throughout all defined subgroups [3]. However, a detailed description of abnormal immunological parameters has only been published in one patient presenting with predominant hypogammaglobulinemia who was detected by abnormal “κ-deleting recombination excision circles” (KREC) via newborn screening [4], suggesting NBAS-associated disease to be an inborn error of immunity. A systematic analysis of a characteristic immunophenotype or altered immunological functionality in NBAS-associated disease has remained elusive.

Inborn errors of immunity can present with isolated immune cell dysfunction, but can also be part of a multisystemic presentation as in DiGeorge syndrome [5], ataxia teleangiectasia [6, 7], or in some forms of familial hemophagocytic lymphohistiocytosis (FHL) [8]. Especially with onset in early infancy, they can be life-threatening, emphasizing the need for early disease recognition, clinical awareness, and high throughput genetic testing [9], but also mechanistic understanding to improve patient management and care [10].

In this study, we systematically determine key immunological parameters in fifteen individuals with confirmed NBAS-associated disease including the cellular composition and function of leukocytes in peripheral blood, immunoglobulin levels, and cytokine gene expression. We reveal that NBAS-associated disease displays a common immunological signature of impaired humoral adaptive immunity and a defective NK cell compartment and, therefore, should be considered an inborn error of immunity.

## Patients and Methods

### Patient Recruitment, Controls, and Data Acquisition

Fifteen patients with biallelic pathogenic variants in *NBAS* from all three subgroups (ILFS2, SOPH, ILFS2-SOPH) were included in this prospective observational study. All procedures were in accordance with the ethical standards of the responsible committee on human experimentation and with the Helsinki Declaration of 1975, as revised in 2013. Informed consent to participate in the study was obtained from all patients or from their parents in the case of minor patients. The study was approved by the ethical committee of the University Hospital Heidelberg and local ethical committees.

The general clinical parameters included country of origin, sex, and age at onset of clinical symptoms. For clinical phenotyping, human phenotype ontology terms were used (see Supplemental Material). For immunological phenotyping, blood from all individuals was drawn outside episodes of inflammation. If more than one measurement per patient was performed, the arithmetic mean was calculated. For flow cytometry and gene expression analysis, gender-matched and age-matched healthy donors were included. For NK cell degranulation assays additional blood samples from six patients were obtained and gender-matched healthy donors were recruited. Both recruitments took place at the University Hospital Heidelberg. Database was closed on December 1, 2019.

### Gene Expression Analysis

Heparinized whole blood of 0.5 ml was incubated with 0.1 ml RPMI1640 or stimulated in RPMI1640 with 100 ng/ml PMA and 5 μg/ml ionomycin (Sigma) for 3 h at 37 °C and 5% CO_2_. Red cells were lysed twice with ACK buffer. Afterward, the leukocytes were lysed with 400 μl of MagNA-Pure lysis buffer (Roche) containing 1% DTT (Roche, cat. no. 10708 984001), and the samples were frozen at − 70 °C. After thawing, the lysates were thoroughly mixed and transferred into the MagNA-Pure sample cartridge and total RNA was isolated with the MagNA-Pure-LC device using the RNA standard protocol for cells. The elution volume was set to 50 μl. An aliquot of 8.2 μl mRNA was reversely transcribed using AMV-RT and oligo-(dT) as primers (First Strand cDNA synthesis kit, Roche) in a thermocycler according to the manufacture’s protocol. After termination of the cDNA synthesis, the reaction mixture was diluted to a final volume of 500 μl and stored at − 20 °C until PCR analysis.

Primer sets optimized for the LightCycler® (RAS) were developed and purchased from SEARCH-LC GmbH (www.Search-LC.com). The PCR was performed with the LightCycler® FastStart DNA Sybr Green kit (RAS) according to the protocol provided in the parameter-specific kits. To control for specificity of the amplification products, a melting curve analysis was performed. The copy number was calculated from a standard curve, obtained by plotting known input concentrations of four different plasmids at log dilutions to the PCR-cycle number (CP) at which the detected fluorescence intensity reaches a fixed value.

To correct for differences in the content of mRNA, the calculated transcript numbers were normalized according to the expression of the housekeeping gene peptidylprolyl isomerase B (PPIB). Values were thus given as transcripts per 1,000 transcripts of PPIB.

### Cytokine Measurement

Cytokine measurement was performed by Myriad RBM (TX, USA) as follows: Using automated pipetting, an aliquot of each sample was added to individual microsphere multiplexes of the selected multi-analyte profile (MAP) and blocker. This mixture was thoroughly mixed and incubated at room temperature for 1 h. Multiplexed cocktails of biotinylated reporter antibodies were added robotically and after thorough mixing, incubated for an additional hour at room temperature. Multiplexes were labeled using an excess of streptavidin–phycoerythrin solution, thoroughly mixed, and incubated for 1 h at room temperature. The volume of each multiplexed reaction was reduced by vacuum filtration and washed 3 times. After the final wash, the volume was increased by addition of buffer for analysis using a Luminex instrument, and the resulting data was interpreted using proprietary software developed by Myriad RBM. For each multiplex, both calibrators and controls were included on each microtiter plate. Eight-point calibrators to form a standard curve were run in the first and last column of each plate and controls at 3 concentration levels were run in duplicate. Study sample values for each of the analytes were determined using 4 and 5 parameter logistics, with weighted and non-weighted curve fitting algorithms included in the data analysis package.

### PBMC Isolation and Culture

Peripheral blood mononuclear cells (PBMCs) were isolated from unfractionated peripheral blood by Ficoll-Hypaque density gradient (density 1.077 g/ml, Biochrom, VWR) centrifugation. For functional and phenotypic analyses, PBMCs were resuspended in SCGM medium (CellGenix) containing 10% human serum (pooled human AB serum, # P041702, Pan-Biotech) and cultured for 2 days without or with 400 U/ml of IL-2 (kindly provided by the NIH, USA).

### Degranulation

CD107a upregulation on NK cells of NBAS-deficient patients or healthy controls was measured after co-culture of NK cells without or with the erythroleukemic cell line K562 (ATCC) at 1:1 ratio (each 5 × 10^4^ cells) in duplicate for 4 h in the presence of anti-CD107a FITC (clone A4A3, Biolegend) or anti-CD107a PE (clone A4A3, Biolegend, and GolgiPlug, 1/100 v/v, BD Bioscience) in 96-well plates in RPMI 1640 (Invitrogen) supplemented with heat-inactivated 10% FCS (Invitrogen) and 1% penicillin/streptomycin (Sigma-Aldrich).

### Flow Cytometry

The immunophenotyping was performed in whole blood. Absolute cell count was measured using the BDMultitest™ 6-color TBNK reagent with BD Trucount™tubes (BD Biosciences, Heidelberg, Germany). The panels are modified recommendations of the “Human Immunophenotyping consortium” [11]. Briefly, Panel-1 consisted of 17 antibodies to characterize the major T cell populations, including naïve and various memory/effector populations (CD2; CD3; CD4; CD8; TCR ƴδ; CD45RA; CD197 (CCR7); CD28; CD57 and CD127). Th1, Th2; Th17, and Tfh cells were identified with the following antibodies: CD183 (CXCR3); CD185 (CXCR5); and CD196 (CCR6). Activation was monitored with antibodies against HLA-DR, CD38, CD278 (ICOS), and CD279 (PD1). Using CD3 as a backbone for absolute quantification, with this comprehensive panel more than 200 defined subpopulations have been quantified in absolute numbers as well as in various ratios. Panel-2 consists of 8 parameters (CD3; CD19; CD20; anti-IgD; CD27; CD10; CD24; and CD38), allowing the identification of transitional, naïve, and memory B cells as well as circulating plasmablasts both in absolute numbers (CD19 backbone) and in various ratios. Panel-3 identified various NK, monocyte and DC subsets and included antibodies with the following specificities: CD11c; CD123; CD14; CD16; CD19; CD2; CD20; CD25; CD3; CD45; CD56; CD57; CD8a; HLA-DR; and M-DC8. Using NK cells as backbone, absolute numbers were calculated for 50 different populations. Finally, the Treg subset was characterized using the following antibodies: CD127; CD194 (CCR4); CD25; CD3; CD4; CD45; CD45RO; and anti-HLA-DR. CD4 + T-lymphocytes served as backbone for the absolute quantification of the different subsets. The analysis was performed on an LSR Fortessa Analyzer (Special Order Research Product) (BD Biosciences). The detailed setup, staining, gating, and analysis procedures have been recently described [12]. Cell surface expression of CD107a and CD69 (marker for NK cell activation) and intracellular expression of perforin and granzyme B of CD3^−^ CD56^+^ NK cells in PBMCs was measured by flow cytometry (LSR Fortessa II, BD Bioscience) and analyzed with FlowJo 10 software (FlowJo LLC). Intracellular staining was performed using the “Cytofix/Cytoperm” kit (BD Bioscience). (For information on antibodies, see Supplemental Material.)

### Statistics

Statistical analyses were performed with GraphPad Prism. Mean values of two groups were compared by Mann–Whitney *U*-tests. Mean values of multiple groups were assessed by one-way ANOVA followed by multiple comparisons post hoc tests as indicated. Error bars represent the SEM and asterisks indicate the significance levels (*P < 0.05; **P < 0.01; ***P < 0.001). The correlation coefficient *R*^2^ was calculated by linear regression.

## Results

### Genotype and Clinical Phenotype

Fifteen patients from 14 unrelated families carrying 19 different *NBAS* variants (Supplemental Fig. 1A) were included in this study. All three NBAS deficiency subgroups were represented (“β-propeller” *n* = 4; “Sec39” *n* = 5; “C-terminal *n* = 4”; not attributable to one of the subgroups *n* = 2) (Table 1). Clinical presentations of 14 of those patients have been reported previously [3, 4], whereas individual NBAS 88 has not been reported before. Thirteen patients originated from European countries, one patient from the USA, and one from Saudi Arabia. The median age at last assessment was 7 years (range: 0.4–43 years). First symptoms occurred in all patients within the first 2 years of life and consisted of acute liver failure or elevated liver transaminases in most cases (73%). Only individual NBAS 88 was diagnosed prenatally and showed immunological laboratory abnormalities and slightly elevated liver transaminases at 8 months of age when the database was closed.

**Table 1:** Genotypes and clinical phenotype

PID	Subgroup	Age at last visit (years)	Descent	Genotypic features				Affected organ systems						
				Allele 1 (nucleotid change; NM_015909.3)	Allele 1 (protein change; NP_056993.2)	Allele 2 (nucleotid change; NM_015909.3)	Allele 2 (protein change; NP_056993.2)	Immune system	Liver	Nervous system	Skeletal system	Growth	Integument	Musculature
NBAS 1	β-propeller	23	PL	c.(558_560del)	p.(Ile187del)	c.(686dupT)	p.(Ser230Glnfs*4)	Y	Y	Y	Y	Y	Y	N
NBAS 7		17	IE	c.(1241C > T)	p.(Ser414Phe)	c.(2950delA)	p.(Ile984Leufs*8)	Y	Y	Y	Y	Y	Y	Y
NBAS 8		5	DE	c.(1241C > T)	p.(Ser414Phe)	c.((6236 + 1_6237-1)_ (6432 + 1_6433-1)del)	p.(Glu2080*)	Y	Y	Y	Y	Y	Y	Y
NBAS 10		17	DE	c.(1278A > C)	p.(Cys426Trp)	c.((4582 + 1_4583-1)_ (4797 + 1_4798-1)del)	p.(Val1528Glyfs*2)	Y	Y	N	Y	Y	Y	N
NBAS 87		1	PK	c.(1948C > T)	p.(Pro650Ser)	c.(1948C > T)	p.(Pro650Ser)	Y	Y	Y	Y	n.d	N	N
NBAS 2	Sec39	28	DE	c.(2708 T > G)	p.(Leu903Arg)	c.(2708 T > G)	p.(Leu903Arg)	Y	Y	Y	Y	N	N	N
NBAS 4		43	DE	c.(2708 T > G)	p.(Leu903Arg)	c.(2827G > T)	p.(Glu943*)	Y	Y	Y	Y	Y	Y	N
NBAS 54		7	SA	c.(2819A > C)	p.(His940Pro)	c.(2819A > C)	p.(His940Pro)	Y	Y	N	N	N	N	N
NBAS 60		2	DE	c.(3534C > A)	p.(Ser1178Arg)	c.(1342-6A > G)	p.(?)	Y	Y	Y	N	N	N	N
NBAS 88*		0.7	DE	c.(3534C > A)	p.(Ser1178Arg)	c.(1342-6A > G)	p.(?)	Y	Y	N	N	N	N	N
NBAS 28	C-terminal	8	ES	c.(5741G > A)	p.(Arg1914His)	c.(2032C > T)	p.(Gln678*)	Y	Y	Y	Y	Y	Y	N
NBAS 29		8	DE	c.(5741G > A)	p.(Arg1914His)	c.(2827G > T)	p.(Glu943*)	Y	Y	Y	Y	Y	Y	Y
NBAS 14		3	HR	c.(5761G > C)	p.(Ala1921Pro)	c.(686dupT)	p.(Ser230Glnfs*4)	Y	Y	N	N	N	N	N
NBAS 57		6	US	c.(5547delC)	p.(Trp1850Glyfs*32)	c.(6966_6969delinsTC)	p.(Gln2322Hisfs*18)	Y	Y	Y	N	N	N	N
NBAS 3		24	DE	c.(603_605del)	p.(Leu202del)	c.(3164 T > C)	p.(Leu1055Pro)	Y	Y	Y	Y	Y	N	N

*PID*, patient identifier; *PL*, Poland; *IE*, Ireland; *DE*, Germany; *PK*, Pakistan; *SA*, Saudi Arabia; *ES*, Spain; *HR*, Croatia; *US*, United States; *Y*, yes; *N*, no; *n.d*., not determined

^*^Family history triggered investigation (brother of NBAS 60); elevated hepatic transaminases as the only symptom of the disease at the age 8 months (closure of study database)

All patients had immunological and hepatic features, while other organ affections were present to different extents according to their NBAS deficiency subgroup (Table 1 [3]). One prominent finding throughout all subgroups was the occurrence of recurrent infections (reported in 10/15 patients). These infections were mostly common with respect to localization and pathogens (Table 2), but intensity suggested a pathological susceptibility in some patients when assessed following the national guideline for clinical diagnostics of inborn errors of immunity [13].

**Table 2:** Immunological phenotype

PID	Subgroup	Recurrent infections (Y/N)	Sepsis (Y/N)	Type of infection (pathogen)	Splenomegaly (Y/N)	Reduced NK cell number (Y/N)	Pelger-Huët anomaly (Y/N)	Response to vaccination (Y/N)	Low IgM (Y/N)	Low IgG (Y/N)	Low IgA (Y/N)	Immunotherapy (N / specific information)
NBAS 1	β-propeller	Y	N	Pneumonia, recurrent urinary tract infections, tonsillitis, gastroenteritis, recurrent conjunctivitis	N	Y	N	N (MMR)	N	N	N	N
NBAS 7		Y	Y	Recurrent pneumonia (RSV, H1N1), sepsis (coagulase-negative staphylococcus), recurrent viral infections	N	Y	Y	N (TD, P)	Y	Y	Y	Intravenous immunoglobulin on a regular basis
NBAS 8		Y	N	Otitis media, gastroenteritis (rotavirus)	N	N	Y	N (TD, MMR)	N	N	Y	N
NBAS 10		N	N		Y	Y	Y	Y	N	Y	Y	N
NBAS 87		Y	Y	Recurrent bacterial infections including urinary tract infections and sepsis, potentially associated with a central intravenous access	N	Y	N	n.a	Y	Y	Y	Intravenous immunoglobulin on a regular basis; steroid therapy
NBAS 2	Sec39	Y	Y	Pharyngitis, rhinitis, tonsillitis, otitis media, gastroenteritis, pneumonia	Y	Y	N	Y	N	N	N	N
NBAS 4		N	m.d	Gastroenteritis	Y	Y	N	Y	N	N	N	N
NBAS 54		Y	Y	Otitis media, mastoiditis, meningitis, gastroenteritis (salmonella), pneumonia (rubella), varicella zoster reactivation on the left leg	N	Y	N	Y	N	N	N	Interferon beta 1a (begun before diagnosis of NBAS deficiency has been made); no hospital admissions or major infections during one year course of treatment; after discontinuation fulminant liver crisis triggered by a febrile upper respiratory tract infection
NBAS 60		N	N	Hepatitis (HHV6)	N	Y	m.d	N (D)	Y	Y	N	Intravenous immunoglobulin once during episode of acute liver failure
NBAS 88*		N	N		N	N	m.d	Y	N	N	Y	N
NBAS 28	C-terminal	Y	Y	Endocarditis, otitis media	N	N	Y	Y	Y	Y	Y	Intravenous immunoglobulin on a regular basis
NBAS 29		Y	N	Respiratory infections (*Candida Albicans*, herpes simplex virus)	N	Y	Y	Y	Y	Y	Y	Subcutaneous immunoglobulin on a regular basis
NBAS 14		Y	N	Respiratory infections, otitis media, gastroenteritis	N	N	Y	m.d	Y	Y	N	N
NBAS 57		Y	N	Pneumonia	N	Y	N	N	Y	Y	Y	Subcutaneous immunoglobulin on a regular basis
NBAS 3		N	N	Otitis media	Y	Y	N	Y	N	N	N	N

*PID*, patient identifier; *Y*, yes; *N*, no; *Ig*, immunoglobulin; *HHV6*, human herpesvirus 6; *MMR*, measles mumps rubella; *TD*, tetanus diphteria; *P*, poliomyelitis; *m.d.*, missing data; *n.a.*, not applicable

^*^Family history triggered investigation (brother of NBAS 60); elevated hepatic transaminases as the only symptom of the disease at the age 8 months (closure of study database); for exact values, see Table S1

### Immunoglobulins Are Reduced in NBAS-Deficient Patients

To assess adaptive humoral abnormalities in NBAS-deficient patients, we examined serum immunoglobulin (Ig) levels. Ig levels of classes M, G, and A were reduced in 10 of the 15 patients, with consistently low IgG being most prevalent (*n* = 8; age at diagnosis: 2 months until 7 years, median: 7.5 months; Table 2). All patients from the “C-terminal” subgroup had low IgG. Six patients with consistently low IgG received Ig replacement therapy, resulting in a reduction of infections. Five patients showed insufficient responses to vaccination when tested sporadically, but also after scheduled vaccinations in 3 patients, pointing to an impaired adaptive humoral immune responsiveness (Table 2). Comparing the B cell phenotype of those patients with impaired Ig levels of all tree classes with those patients with normal levels of Ig, the relative number of transitional B cells seems to be increased (22.22 ± 10.44 vs. 6.73 ± 3.73; *P* = 0.034), pointing to delayed B cell maturation in the patients with humoral immunodeficiency. Differences in B cell subpopulations between patients with specific Ig isotype deficiencies could not be observed.

### CD56^+^ NK Cells and CD19^+^-Naïve B Cells Are Reduced in NBAS-Deficient patients

**Fig. 1:**
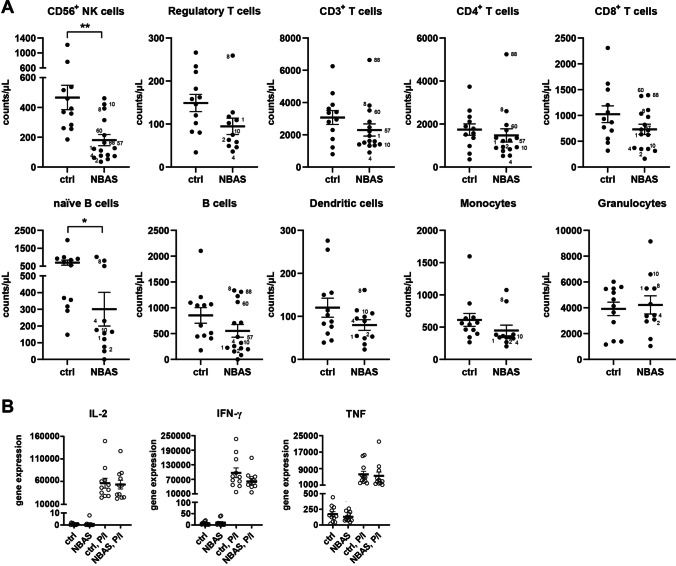
Circulating CD56 + NK cells are reduced in NBAS patients. **B** Absolute cell numbers of leukocytes subsets per microliters of peripheral blood were quantified in fifteen NBAS patients relative to a group of twelve healthy controls by flow cytometry. Indexed patients were tested throughout this study in phenotypic and functional assays. **B** Gene expression was determined in whole blood of NBAS patients or healthy controls in steady state or after 3-h stimulation with PMA and ionomycin

To analyze the composition of cellular immunity, peripheral blood leukocyte subsets were quantified. The majority of NBAS-deficient patients showed a consistent reduction in CD56^+^ NK cells, resulting in > 50% lower absolute NK cell numbers relative to healthy controls (Fig. 1A–B and Supplemental Fig. 1B). This reduction in CD56^+^ NK cells was maintained over time in 8 of 15 patients, who could be assessed at two to five time points over a period of 8 to up to 1100 days after the first visit/measurement (Supplemental Fig. 1C). Naïve CD19^+^ B cell numbers were considerably lower and correlated with the reduction in NK cells in most patients. Frequencies of other leukocyte subsets or B cell subsets were reduced to a lower extent or were heterogeneous (Fig. 1B, Supplemental Fig. 1B and D).

Next, the gene expression status of immune cell–secreted molecules in leukocytes (no subsets) was measured. The expression of key cytokines such as IL-2, IFN-γ, and TNF was not altered in steady state or after stimulation with PMA/ionomycin compared to healthy controls. Blood cytokine levels of IL-2, IFN-γ, and TNF did not differ between patients and controls (data not shown). Altogether, the quantification of leukocyte subsets revealed a significant reduction in NK cells and naïve B cells as the most consistent cellular biomarker of immune deficiency.

### The CD56DIM CD16 + NK Cell Subset Is Reduced in NBAS-Deficient Patients

**Fig. 2:**
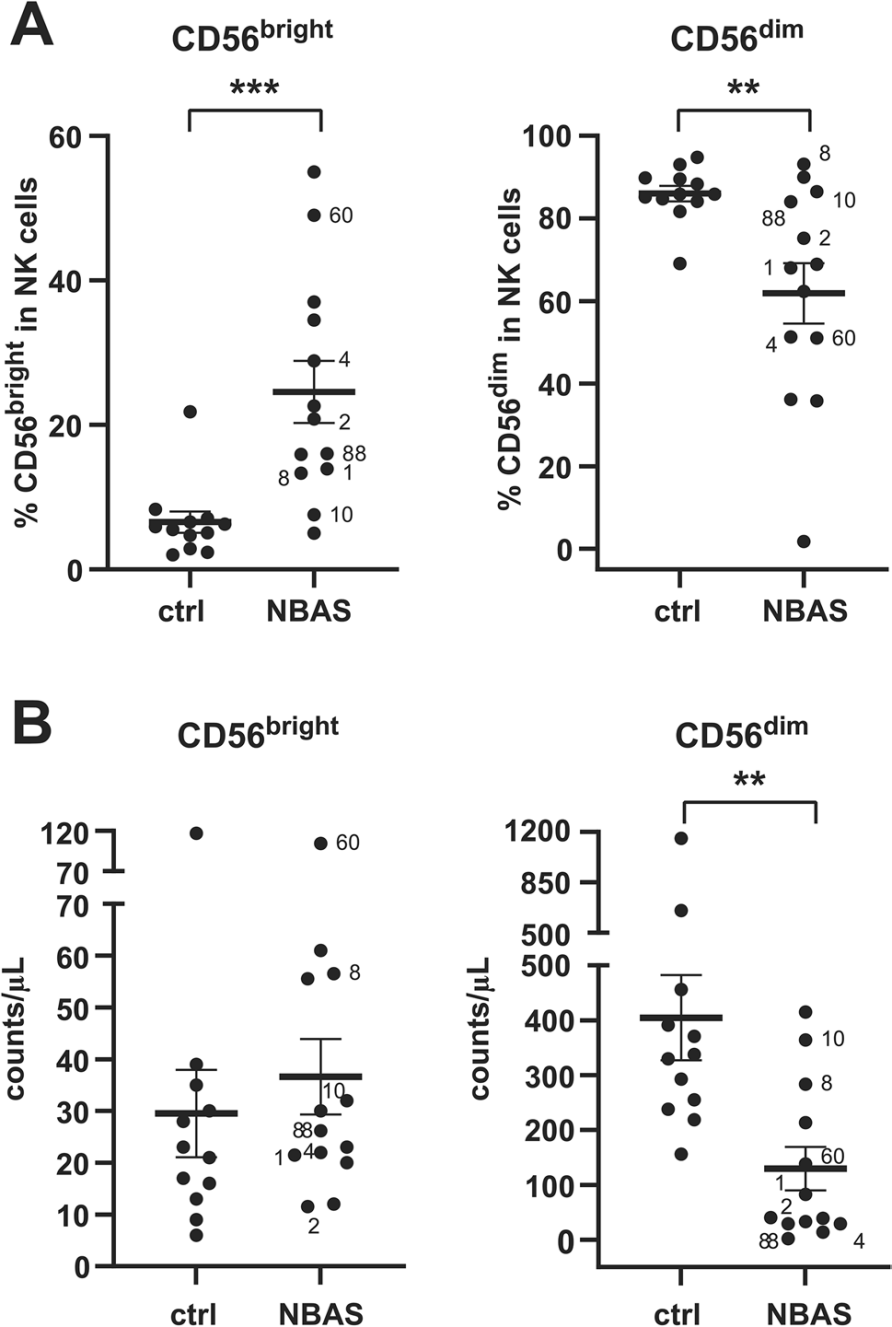
The reduction in NK cells is due to the low numbers of the CD56^dim^ NK cell subset. **A** The percentage of CD56^bright^ and CD56^dim^ NK cell subsets within CD56^+^ NK cells was measured by flow cytometry. **B** Absolute cell numbers per microliters of peripheral blood of CD56^bright^ and CD56^dim^ NK cell subsets

To extend the phenotypic characterization of NK cells in NBAS-deficient patients, we analyzed the percentage of NK cell subsets within PBMCs. The percentage of the CD56^dim^ CD16^+^ NK cell subset was significantly reduced in NBAS-deficient patients compared to healthy controls (Fig. 2A). The decrease of the percentage of CD56^dim^ CD16^+^ NK cells was paralleled by the reduction of the absolute cell number of the CD56^dim^ CD16^+^ NK cell subset, whereas the absolute cell number of the CD56^bright^ CD16^−^ NK cell subset was unchanged (Fig. 2B). Hence, the reduction in NK cells in PBMCs of NBAS-deficient patients was a consequence of the reduction in the CD56^dim^ CD16^+^ NK cell subset.

### NBAS-Deficient NK Cells Are Hyporesponsive to Target Cell Stimulation

*NBAS* transcripts have been detected in numerous human tissues including blood [14], and NBAS protein was detected in primary NK cells and NK cell lines (Supplemental Fig. 1E); hence, NBAS deficiency could have an intrinsic effect on NK cell biology.

**Fig. 3:**
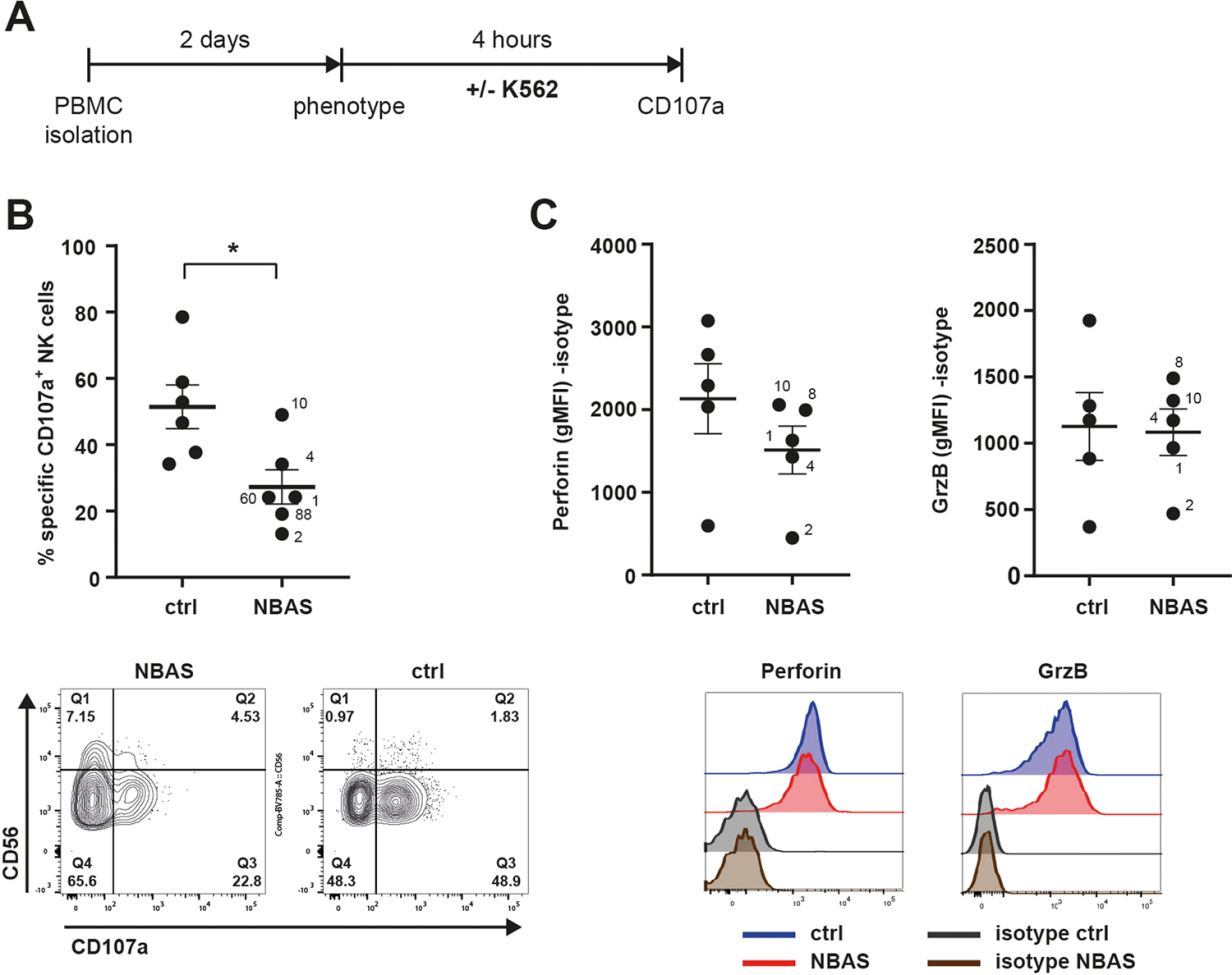
Residual NK cells in NBAS patients are functionally impaired upon activation by K562 cells. **A** Scheme of the experimental setup. **B** NK cell degranulation, indicated by the percentage of CD107a^+^ cells of CD56^+^ NK cells, was measured by flow cytometry after 4-h co-culture of NBAS patients’ or healthy controls’ NK cells with K562 target cells. Baseline CD107a^+^ in the absence of K562 cells was subtracted. Combined data, comparing the mean of six patients to that of healthy controls, and representative FACS contour plots of one patient and healthy control combination are depicted (limits were set according to CD107a expression in the absence of K562 cells and distinguishing CD56^bright^ and CD56^dim^ NK cell subsets). **C** Intracellular protein expression of perforin and granzyme B in NK cells, which had not been co-cultured with K562 target cells, was measured by flow cytometry (geometric mean fluorescence intensity, gMFI). Isotype control staining was subtracted. Combined data, comparing the mean of five patients to that of healthy controls, and representative FACS histograms of one patient (red line) and healthy control (blue line) combination are depicted

We investigated whether the remaining NK cells in NBAS-deficient patients retained responsiveness to the prototypical target cell line K562 by measuring CD107a upregulation on NK cells as a functional marker for cytolytic granule release (Fig. 3A–B). For this functional analysis, blood samples could be obtained from six patients that belonged to the “β-propeller” and the “Sec39” subgroup, but not from patients from the “C-terminal” subgroup. CD107a upregulation on NK cells was tested outside inflammatory episodes or liver decompensations. The mean percentage of CD107a^+^ NK cells of NBAS-deficient patients in response to K562 cells was considerably decreased, resulting in an approx. 50% reduction in CD107a upregulation as compared to NK cells from healthy controls (Fig. 3B). The reduction in CD107a upregulation was apparent in both the CD56^dim^ and the CD56^bright^ NK cell populations. Accordingly, the reduction in peripheral blood NK cell numbers was accompanied by a reduced responsiveness of the NK cells in NBAS-deficient patients. Intracellular perforin protein expression in NK cells was lower in NBAS-deficient patients but did not reach statistical significance, while granzyme B levels were unchanged (Fig. 3C). In conclusion, the reduced NK cell population in NBAS-deficient patients appeared to be less responsive to target cell stimulation in terms of degranulation.

### IL-2 Activation Rescues the Responsiveness of NBAS-Deficient Patients’ NK Cells

**Fig. 4:**
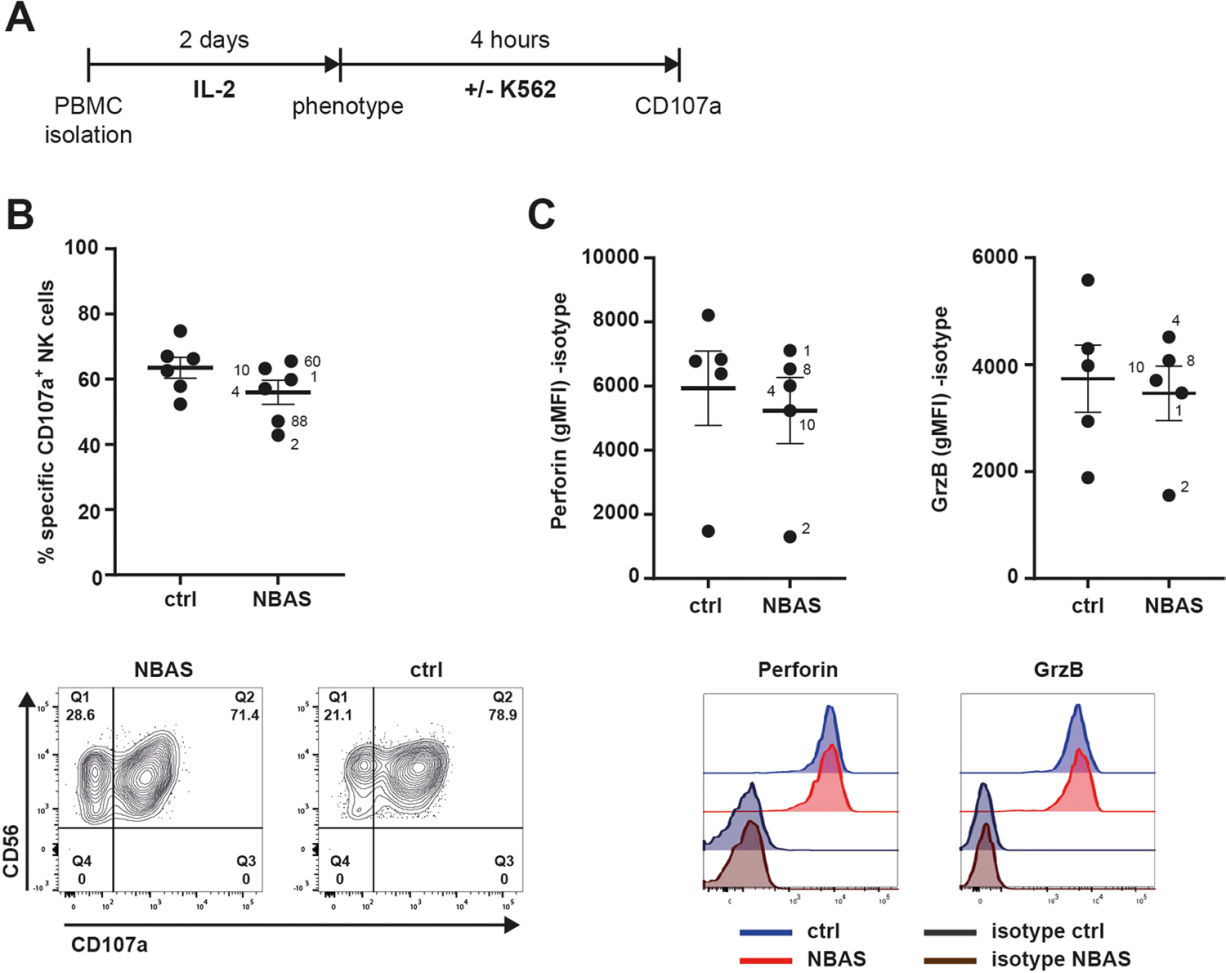
NK cell dysfunction of NBAS patients can be rescued by pre-activation with IL-2. **A** Scheme of the experimental setup. **B** NK cell degranulation, indicated by the percentage of CD107a^+^ cells of CD56^+^ NK cells, was measured and analyzed as described in Fig. 3. In the representative FACS contour plots, limits were for total CD56^+^ NK cells due to blurred distinction of CD56^bright^ and CD56^dim^ NK cell subsets upon IL-2 treatment. **C** Intracellular protein expression of perforin and granzyme B in NK cells was determined and analyzed as described in Fig. 3

Next, we assessed whether functionality of patients’ NK cells can be improved by the NK cell-activating cytokine IL-2 (Fig. 4A) in vitro. Exposure to IL-2 for 2 days induced upregulation of the early activation marker CD69 on the cell surface of NBAS-deficient NK cells, but still to a lower extend than observed in healthy control NK cells (Supplemental Fig. 2A). Importantly, IL-2-exposed NK cells of NBAS-deficient patients showed enhanced CD107a upregulation in response to K562, which was similar to that of IL-2-exposed NK cells from healthy controls (Fig. 4B). Activation by IL-2 substantially improved NK cell degranulation even of those patient cells which were very weak responders to K562 without IL-2 pre-activation (i.e., patients 2, 60, and 88). Congruent with the higher activation potential, patients exhibiting high CD107a upregulation also showed higher CD69 expression (Supplemental Fig. 2B). Moreover, intracellular perforin and granzyme B protein expression levels were increased and similar between IL-2-exposed NK cells of NBAS-deficient patients and healthy controls (Fig. 4C). Thus, IL-2 cytokine-induced activation can rescue the functionality of NBAS-deficient patient NK cells and their responsiveness to target cells.

## Discussion

NBAS-associated disease is a complex disorder with a heterogeneous clinical spectrum involving multiple organ systems that can be categorized into three different clinical subgroups “β-propeller,” “Sec39,” and “C-terminal” [3]. Abnormalities of the immune system are present in a majority of NBAS-deficient individuals of all three subgroups [3], but no detailed characterization of this affection has been done so far. In this study, we demonstrate that the immunodeficiency phenotype of NBAS-associated disease is characterized by quantitative and qualitative NK cell and B cell deficiency. The lower NK cell counts correlated with the loss of the more mature CD56dim CD16^+^ cytotoxic NK cell subset, pointing toward dysfunctional NK cell maturation and/or disrupted mature NK cell homeostasis. The residual NK cell population exerted a significantly lower potential for activation and degranulation upon contact with K562 target cells. This hyporesponsive state could be rescued in vitro by pre-activation with the NK cell-stimulating cytokine IL-2. Due to a lack of available biosamples, functional analysis of NK cells was tested in patients from the “β-propeller” and the “Sec39” subgroup, but not in patients from the “C-terminal” group.

Altered NK cell development and numbers of NK cells and ensuing susceptibility to herpes or papilloma virus infections are hallmarks of classical NK cell deficiencies [15]. Importantly, patients with NBAS-associated disease suffer from frequent and recurrent infections (Table 2 and authors’ unpublished data) such as human herpesvirus 6-associated severe crisis of NK cell depletion and liver failure in individual NBAS 60. In addition, susceptibility of NBAS-deficient patients to viral infections may also be facilitated by defective humoral immunity, since consistently low IgG serum levels and partially impaired specific antibody generation after vaccination were prevalent in these patients [3].

Among classical NK cell deficiencies, the most frequently cited defect, GATA2 deficiency, affects CD56^bright^ NK cells [16], while NK cell deficiencies due to variants in *IRF8* and *MCM4* mainly affect CD56^dim^ NK cells and present with impaired cytolytic function, similar to the pattern observed in our patients [17, 18].

Dysfunctional NK cell degranulation is one of the diagnostic parameters of hemophagocytic lymphohistiocytosis (HLH) which is a life-threatening hyperinflammatory syndrome. HLH has been categorized into primary FHL, syndromic HLH, and secondary HLH mainly driven by infections. Primary HLH itself is characterized by uncontrolled activation of lymphocytes and macrophages due to impaired cytotoxic function of NK cells and T cells, leading to excessive cytokine production like IFN-γ [19]. While the cytotoxic potential of NK cells in NBAS-deficient patients from the “β-propeller” and “Sec39” subgroup was likewise reduced, we did not observe increased pro-inflammatory cytokine levels in serum of NBAS-deficient patients in remission. However, no data on cytokine levels or the status of myeloid cell activation during inflammatory states or, of specific interest, during episodes of liver failure were available.

Systemic hyperinflammation in the context of HLH is a major cause of acute liver failure in the first year of life [20, 21]. However, there is growing evidence that dysregulated hepatic inflammation per se can lead to acute liver failure [22, 23] and NBAS-deficient patients from the “β-propeller” and “Sec39” subgroup have fever-related acute liver failure [3]. As elevated temperature was shown to cause a reduction in NBAS protein level and cell viability [24], fever might affect NK cell homeostasis and viability particularly in NBAS-deficient cells. In line with this is the observation that absolute NK cell numbers further decreased in individuals NBAS 8 and NBAS 60 under the level of detection during febrile episodes (data not shown). However, NK cells were low in number and dysfunctional also outside episodes of inflammation or hepatic dysfunction. There may be evolution of the immune phenotype in NBAS-associated over life time, which could be one explanation for the immune-phenotypic inconsistency among siblings sharing the same genotype (NBAS 60, NBAS 88). Further studies are needed to study NK cell activation, cell signaling of the JAK-STAT pathways [23], cytokine secretion, such as IL-18, and IL-18BP [22], and degranulation in NBAS deficiency especially during fever-triggered acute liver failure. This might set the stage of targeted treatment of NBAS deficiency associate acute liver failure, e.g., with Janus kinase inhibitors [25] or IL-18 binding protein (ClinicalTrials.gov Identifier: NCT03113760).

Apart from a hepatic phenotype, the FHL, and syndromic HLH can include growth retardation, neurological dysfunction [26–28] and hypogammaglobulinemia (FHL5 [29], Griscelli syndrome type 2 [20, 30], XLP-1 deficiency, and XPL-2/XIAP deficiency [31]), all of which are prevalent in NBAS deficiency [3]. Thus, HLH and NBAS-associated disease share clinical symptoms and immunological abnormalities (Table 3).

**Table 3:** FHL and HLH syndromes: genes, functions, and cytometric abnormalities

	Disease name	Phenotype MIM number(s)	Affected gene	Affected cellular function	Flow cytometric abnormality
FHL	FHL1	267700	Unknown	Unknown	Not available
	FHL2	603553	*PRF1*	Pore formation	Decreased / absent perforin expression
	FHL3	608898	*UNC13D*	Vesicle priming	Decreased CD107a expression
	FHL4	603552	*STX11*	Vesicle fusion	Decreased CD107a expression
	FHL5	601717	*STXBP2*	Vesicle fusion	Decreased CD107a expression
HLH syndromes	Griscelli syndrome type 2	607624	*RAB27A*	Vesicle docking	Decreased CD107a expression
	Chediak-Higashi syndrome	214500	*LYST*	Vesicle trafficking	Decreased CD107a expression
	Hermansky-Pudlak syndrome 2	608233	*AP3B1*	Vesicle trafficking	Decreased CD107a expression
	Hermansky-Pudlak syndrome 9	614171	*BLOC1S6*	Vesicle trafficking	Decreased CD107a expression
	NBAS-associated disease (ILFS2, SOPH)	616483; 614800	*NBAS*	Vesicle trafficking	Decreased CD107a expression

*FHL*, familial hemophagocytic lymphohistiocytosis; *HLH*, hemophagocytic lymphohistiocytosis; *ILFS2*, infantile liver failure syndrome type 2; *SOPH*, short stature, optic nerve atrophy, Pelger-Huët anomaly

FHL2-5 and syndromic HLH share a pathophysiology of defective biogenesis and trafficking of cytolytic vesicles including their priming and fusion with the plasma membrane (Table 3 [8]). The Sec39 domain of NBAS is part of the syntaxin 18 (STX18) complex, a soluble *N*-ethylmaleimide-sensitive-factor attachment receptor (SNARE) complex involved in anterograde and retrograde transport between endoplasmic reticulum and Golgi [2, 24, 32, 33]. Additionally, STX18 has been shown to directly interact with syntaxin 4 (STX4), a known mediator of degranulation of cytolytic vesicles that is located at the plasma membrane [34, 35]. While degranulation activity of NK cells is decreased in NBAS-deficient patients, expression levels of granzyme B and perforin remain widely unchanged. We speculate that impaired STX18 complex function in NBAS-associated disease might impair NK cell cytotoxicity by disturbing granule release of cytotoxic vesicles. More globally and since STX4 is known to be involved in IgG exocytosis, insulin secretion and glucose uptake in the musculature and adipose tissue, a dysfunctional interaction between STX4 and STX18 might be a driver not only for hypogammaglobulinemia but also for diabetes mellitus type 1, muscular hypotonia, and reduced subcutaneous fat tissue, which are all found in NBAS-associated disease [36]. Other diseases affecting STX18 complex function such as RINT1 deficiency, which is associated with recurrent liver failure and skeletal abnormalities and hence shows phenotypic overlap to NBAS deficiency, should be investigated for trafficking of cytolytic vesicles and impaired NK cell cytotoxicity [37].

We show that NK cell activation and degranulation were improved by pre-activation of NK cells with IL-2 in vitro in patients from the “β-propeller” and “Sec39” subgroup, suggesting that IL-2 might enhance NBAS expression and/or function or promote alternative molecular pathways mediating cytolytic vesicle release in those. Similarly, NK cell degranulation was shown to be improved after 48–72-h IL-2 stimulation in NK cells of FHL4 patients due to variants in *STX11* and FHL5 due to variants in *STXBP2*, with both genes coding for SNARE proteins [29, 38]. Hence, cytokine therapy with clinically approved IL-2 or IL-15 formulations [36, 39, 40] might support NK cell function in NBAS-deficient patients. Clearly, future in-depth mechanistic studies are warranted to better understand the complex pathophysiology of NBAS deficiency.

In conclusion, *NBAS* variants cause a multisystem disease including an immunodeficiency phenotype marked by low NK cell numbers and NK cell dysfunction as well as reduction of naïve B cells and serum immunoglobulin levels. Patients with pathogenic *NBAS* variants should be investigated accordingly.

## Supplementary Information

Below is the link to the electronic supplementary material.

Supplementary file1 (DOCX 359 KB)

## Data Availability

Clinical data files are stored at Heidelberg University Hospital. It may be shared if needed.

## References

[1] Maksimova N, Hara K, Nikolaeva I, Chun-Feng T, Usui T, Takagi M (2010). Neuroblastoma amplified sequence gene is associated with a novel short stature syndrome characterised by optic nerve atrophy and Pelger-Huet anomaly. J Med Genet.

[2] Haack TB, Staufner C, Kopke MG, Straub BK, Kolker S, Thiel C (2015). Biallelic mutations in NBAS cause recurrent acute liver failure with onset in infancy. Am J Hum Genet.

[3] Staufner C, Peters B, Wagner M, Alameer S, Baric I, Broue P, et al. Defining clinical subgroups and genotype-phenotype correlations in NBAS-associated disease across 110 patients. Genetics in medicine: official journal of the American College of Medical Genetics. 2020;22(3):610–21. 10.1038/s41436-019-0698-4 31761904

[4] Ricci S, Lodi L, Serranti D, Moroni M, Belli G, Mancano G (2019). Immunological features of neuroblastoma amplified sequence deficiency: report of the first case identified through newborn screening for primary immunodeficiency and review of the literature. Front Immunol.

[5] Bassett AS, McDonald-McGinn DM, Devriendt K, Digilio MC, Goldenberg P, Habel A, et al. Practical guidelines for managing patients with 22q11.2 deletion syndrome. J Pediatr. 2011;159(2):332–9. 10.1016/j.jpeds.2011.02.039 PMC3197829 21570089

[6] Gatti RPS. Ataxia-Telangiectasia. Seattle (WA): University of Washington, Seattle1999 Mar 19 [Updated 2016 Oct 27]. Available from: https://www.ncbi.nlm.nih.gov/books/NBK26468/. Accessed 02 Apr 2021.

[7] Nissenkorn A, Levy-Shraga Y, Banet-Levi Y, Lahad A, Sarouk I, Modan-Moses D (2016). Endocrine abnormalities in ataxia telangiectasia: findings from a national cohort. Pediatr Res.

[8] Chandrakasan S, Filipovich AH (2013). Hemophagocytic lymphohistiocytosis: advances in pathophysiology, diagnosis, and treatment. J Pediatr.

[9] King JR, Hammarström L (2018). Newborn screening for primary immunodeficiency diseases: history, current and future practice. J Clin Immunol.

[10] Bonilla FA, Khan DA, Ballas ZK, Chinen J, Frank MM, Hsu JT, et al. Practice parameter for the diagnosis and management of primary immunodeficiency. J Allergy Clin Immunol. 2015;136(5):1186–205.e1–78. 10.1016/j.jaci.2015.04.049 26371839

[11] Maecker HT, McCoy JP, Nussenblatt R (2012). Standardizing immunophenotyping for the human immunology project. Nat Rev Immunol.

[12] Oras A, Quirant-Sanchez B, Popadic D, Thunberg S, Winqvist O, Heck S (2020). Comprehensive flow cytometric reference intervals of leukocyte subsets from six study centers across Europe. Clin Exp Immunol.

[13] Diagnostik auf Vorliegen eines primären Immundefekts [Internet]. AWMF. 2017 [cited 22.01.2020]. Available from: https://www.awmf.org/uploads/tx_szleitlinien/112-001l_S2k_Primaere_Immundefekte_PID_2017-11.pdf. Accessed 24 Mar 2021.

[14] Uhlen M, Fagerberg L, Hallstrom BM, Lindskog C, Oksvold P, Mardinoglu A, et al. Tissue-based map of the human proteome. Science. 2015;347(6220):1260419. 10.1126/science.1260419 25613900

[15] Orange JS (2013). Natural killer cell deficiency. J Allergy Clin Immunol.

[16] Mace EM, Hsu AP, Monaco-Shawver L, Makedonas G, Rosen JB, Dropulic L (2013). Mutations in GATA2 cause human NK cell deficiency with specific loss of the CD56(bright) subset. Blood.

[17] Gineau L, Cognet C, Kara N, Lach FP, Dunne J, Veturi U (2012). Partial MCM4 deficiency in patients with growth retardation, adrenal insufficiency, and natural killer cell deficiency. J Clin Investig.

[18] Mace EM, Bigley V, Gunesch JT, Chinn IK, Angelo LS, Care MA (2016). Biallelic mutations in IRF8 impair human NK cell maturation and function. J Clin Investig.

[19] Morimoto A, Nakazawa Y, Ishii E (2016). Hemophagocytic lymphohistiocytosis: pathogenesis, diagnosis, and management. Pediatr Int.

[20] Dhawan A, Mieli-Vergani G (2005). Acute liver failure in neonates. Early Human Dev.

[21] Kathemann S, Bechmann LP, Sowa JP, Manka P, Dechene A, Gerner P (2015). Etiology, outcome and prognostic factors of childhood acute liver failure in a German Single Center. Ann Hepatol.

[22] Belkaya S, Michailidis E, Korol CB, Kabbani M, Cobat A, Bastard P (2019). Inherited IL-18BP deficiency in human fulminant viral hepatitis. J Exp Med.

[23] Boehmer DFR, Koehler LM, Magg T, Metzger P, Rohlfs M, Ahlfeld J (2020). A novel complete autosomal-recessive STAT1 LOF variant causes immunodeficiency with hemophagocytic lymphohistiocytosis-like hyperinflammation. J Allergy Clin Immunol Pract.

[24] Staufner C, Haack TB, Kopke MG, Straub BK, Kolker S, Thiel C (2016). Recurrent acute liver failure due to NBAS deficiency: phenotypic spectrum, disease mechanisms, and therapeutic concepts. J Inherit Metab Dis.

[25] O’Shea JJ, Gadina M (2019). Selective Janus kinase inhibitors come of age. Nat Rev Rheumatol.

[26] Henter J-I, Horne A, Aricó M, Egeler RM, Filipovich AH, Imashuku S (2007). HLH-2004: diagnostic and therapeutic guidelines for hemophagocytic lymphohistiocytosis. Pediatr Blood Cancer.

[27] Gray PE, Shadur B, Russell S, Mitchell R, Buckley M, Gallagher K (2017). Late-onset non-HLH presentations of growth arrest, inflammatory arachnoiditis, and severe infectious mononucleosis, in siblings with hypomorphic defects in UNC13D. Front Immunol.

[28] Haddad E, Sulis ML, Jabado N, Blanche S, Fischer A, Tardieu M (1997). Frequency and severity of central nervous system lesions in hemophagocytic lymphohistiocytosis. Blood.

[29] Meeths M, Entesarian M, Al-Herz W, Chiang SCC, Wood SM, Al-Ateeqi W (2010). Spectrum of clinical presentations in familial hemophagocytic lymphohistiocytosis type 5 patients with mutations in STXBP2. Blood.

[30] Pastural E, Barrat FJ, Dufourcq-Lagelouse R, Certain S, Sanal O, Jabado N (1997). Griscelli disease maps to chromosome 15q21 and is associated with mutations in the myosin-Va gene. Nat Genet.

[31] Pachlopnik Schmid J, Cote M, Menager MM, Burgess A, Nehme N, Menasche G (2010). Inherited defects in lymphocyte cytotoxic activity. Immunol Rev.

[32] Aoki T, Ichimura S, Itoh A, Kuramoto M, Shinkawa T, Isobe T (2009). Identification of the neuroblastoma-amplified gene product as a component of the syntaxin 18 complex implicated in Golgi-to-endoplasmic reticulum retrograde transport. Mol Biol Cell.

[33] Raote I, Ortega-Bellido M, Santos AJ, Foresti O, Zhang C, Garcia-Parajo MF, et al. TANGO1 builds a machine for collagen export by recruiting and spatially organizing COPII, tethers and membranes. eLife 2018;7:e32723. 10.7554/eLife.32723. 10.7554/eLife.32723 PMC5851698 29513218

[34] Hatsuzawa K, Tamura T, Hashimoto H, Hashimoto H, Yokoya S, Miura M (2006). Involvement of syntaxin 18, an endoplasmic reticulum (ER)-localized SNARE protein, in ER-mediated phagocytosis. Mol Biol Cell.

[35] Blank U, Madera-Salcedo IK, Danelli L, Claver J, Tiwari N, Sanchez-Miranda E (2014). Vesicular trafficking and signaling for cytokine and chemokine secretion in mast cells. Front Immunol.

[36] Jewell JL, Oh E, Thurmond DC (2010). Exocytosis mechanisms underlying insulin release and glucose uptake: conserved roles for Munc18c and syntaxin 4. Am J Physiol Regul Integr Comp Physiol.

[37] Cousin MA, Conboy E, Wang JS, Lenz D, Schwab TL, Williams M (2019). RINT1 Bi-allelic variations cause infantile-onset recurrent acute liver failure and skeletal abnormalities. Am J Hum Genet.

[38] Bryceson YT, Rudd E, Zheng C, Edner J, Ma D, Wood SM (2007). Defective cytotoxic lymphocyte degranulation in syntaxin-11 deficient familial hemophagocytic lymphohistiocytosis 4 (FHL4) patients. Blood.

[39] Bernatchez C, Haymaker CL, Hurwitz ME, Kluger HM, Tetzlaff MT, Jackson N, et al. Effect of a novel IL-2 cytokine immune agonist (NKTR-214) on proliferating CD8+T cells and PD-1 expression on immune cells in the tumor microenvironment in patients with prior checkpoint therapy. J Clin Oncol. 2017;35(15_suppl):2545.

[40] Rosenberg SA, Yang JC, Topalian SL, Schwartzentruber DJ, Weber JS, Parkinson DR (1994). Treatment of 283 consecutive patients with metastatic melanoma or renal cell cancer using high-dose bolus interleukin 2. JAMA.

