# Supplementary material for: NBAS Variants Are Associated with Quantitative and Qualitative NK and B Cell Deficiency
Source: J Clin Immunol. 2021 Aug 13;41(8):1781–93. doi: 10.1007/s10875-021-01110-7 (PMC8604887; doi:10.1007/s10875-021-01110-7)

**Title: *NBAS* variants are associated with quantitative and qualitative NK cell and B cell deficiency**

**Supllemental DAta**

**Supplemental methods**

**HPO terms for clinical phenotyping**

- Recurrent infections (HP:0002719) including the type of infection
- Sepsis (HP:0100806)
- Splenomegaly (HP:0001744)
- Reduced NK cell number (HP:0040218)
- Pelger-Huët anomaly (HP:0011447)
- Decreased specific antibody response to vaccination (HP:0032140)
- Abnormal immunoglobulin levels of immunoglobulin M (HP:0410243)
- Abnormal immunoglobulin levels of immunoglobulin G (HP:0410242)
- Abnormal immunoglobulin levels of immunoglobulin A (HP:0410240)
- Immunoglobulin replacement therapy (no HP term),

**Analyzed leukocyte subsets**

- CD3^+^ T cells
- CD3^+^ CD4^+^ T cells
- CD3^+^ CD8^+^ T cells
- CD3^+^ CD4^+^ CCR4^+^ CD25^+^ CD127^low^ regulatory T cells
- CD3^-^ CD19^+^ B cells
- CD3^-^ CD19^+^ CD27^-^ naïve B cells
- CD3^-^ CD19^+^ CD27^-^ CD10^+^ immature B cells
- CD3^-^ CD19^+^ CD27^-^ CD24^high^ CD38^high^ transitional B cells
- CD3^-^ CD19^+^ CD27^+^ IgD^+^ pre-switched memory B cells
- CD3^-^ CD19^+^ CD27^+^ IgD^-^ switched memory B cells
- CD3^-^ CD19^+^ CD27^+^ CD38^+^ CD20^-^ plasmablasts
- CD3^-^ CD19^-^ CD14^+^ monocytes
- CD3^-^ CD19^-^ CD14^-^ HLA-DR^+^ dendritic cells
- CD3^-^ CD19^-^ CD14^-^ CD16^-^ CD56^bright^ NK cells
- CD3^-^ CD19^-^ CD14^-^ CD16^+^ CD56^dim^ NK cells
- Granulocytes identified by granularity and size (SSC^high^ FSC^low^)

**Antibodies (BD Bioscience) for FACS staining**

- CD56 BV785 (clone 5.1H11, CD3 APC-Cy7 (clone HIT3a)
- CD45 PerCP-Cy5.5 (clone HI30)
- CD69 PerCP-Cy5.5 (FN50)
- perforin PE (clone BD48)
- Granzyme B Pacific Blue (GB11) (Biolegend)
- CD56 APC (NCAM16.2)
- CD3 PerCP-Cy5.5 (SK7)

**Western blot**

For western blot analyses, isolated NK cells enriched from healthy control PBMCs isolated from buffy coats and cell pellets from the NK cell line NKL were washed in phosphate buffered saline, and resolved in radioimmunoprecipitation assay buffer. Twenty µg of protein of every sample were separated on an 8% polyacrylamide gel. Primary antibodies against NBAS (Merck KGaA, Darmstadt, Germany; rabbit-anti-human; dilution 1:1,000 in tris-buffered saline [0.1% tween, TBST]) and β-actin (Santa Cruz Biotechnology, Heidelberg, Germany; mouse-anti-human; dilution 1:10,000 in TBST [0.1% tween]), were incubated overnight. Secondary hrp-coupled antibodies (goat-anti-rabbit) were from Dianova (Hamburg, Germany) and used in a dilution of 1:10,000 in TBST [0.1% tween]. Enhanced chemiluminescence of proteins was detected using a Vilberscan Fusion FX7.

**Supplemental Tables**

Supplemental Table S1. Minimal NK cell count

| **PID** | **subgroup** | **Reduced NK cell number (Y/N)** | **Minimal NK cell count**  **(NK cells/µl)** |
| --- | --- | --- | --- |
| NBAS 1 | β-propeller | Y | 118 |
| NBAS 7 |  | Y | 53 |
| NBAS 8 |  | N | 161 |
| NBAS 10 |  | Y | m.d. |
| NBAS 87 |  | Y | 81 |
| NBAS 2 | Sec39 | Y | 71 |
| NBAS 4 |  | Y | 76 |
| NBAS 54 |  | Y | 30 |
| NBAS 60 |  | Y | 2 |
| NBAS 88 |  | N | 164 |
| NBAS 28 | C-terminal | N | 461 |
| NBAS 29 |  | Y | 36 |
| NBAS 14 |  | N | 315 |
| NBAS 57 |  | Y | 88 |
| NBAS 3 |  | Y | 111 |

**Abbreviations:** PID, patient identifier; Y, yes; N, no; m.d., missing data

**Figure Legends Supplemental FigureS**

**Supplemental Figure 1. CD56+ NK cells in NBAS patients correlate with naïve CD19+ B cells, and NK cells intrinsically express NBAS protein**

(A) Graphical illustration of the *NBAS* gene and the two known protein domains including the variants of the fifteen studied patients. Top: nonsense and splice site variants as well as deletions of one or more exons. Bottom: missense variants and in-frame deletions. (B) To complement the quantification of leukocyte subsets of figure 1A, the mean of absolute cell numbers of indicated cell subsets of NBAS patients was normalized to the respective mean of healthy controls and calculated as the fold-change. Fold-changes are indicated per leukocyte subset per individual NBAS patient in a tabular heatmap. Shaded in red, fold-changes below 0.5; shaded in yellow, fold-changes between 0.5 and 1.2; shaded in blue, fold-changes above 1.2; n.d., not determined. (C) Quantification of absolute CD56+ NK cell numbers, as described in figure 1, depicting individual measurement time points per patient (left panel) and individual measurements per patient over a period of up to 1100 days after the first visit/measurement (right panel). (D) Quantification of absolute cell numbers of B cell subsets as described in figure 1. (E) Western Blot protein expression of NBAS and β-actin in the NK cell line NKL as well as in primary NK cells enriched from healthy control PBMCs isolated from buffy coats.

**
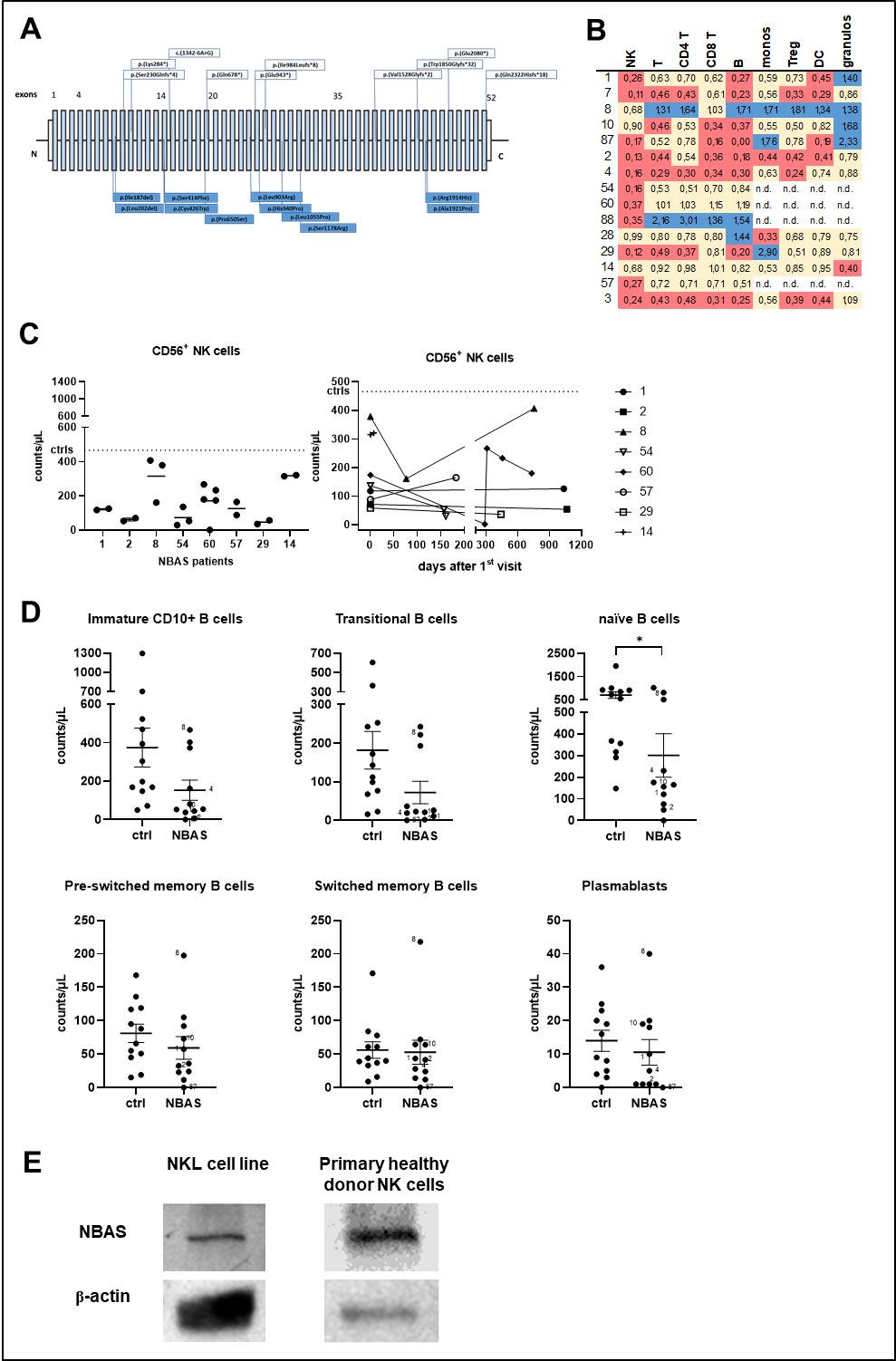
**

**Supplemental Figure 2. NK cells of NBAS-deficient patients up-regulate CD69 upon IL-2 stimulation.**

(A) Surface CD69 expression on NK cells of NBAS patients or healthy controls was measured after two-day culture in medium (i.e., resting NK cells) or IL-2 by flow cytometry. Unspecific isotype control staining was subtracted. Combined data, comparing the mean of five patients to that of healthy controls, and representative FACS histograms and FACS contour plots of one patient and healthy control combination as indicated are depicted. (B) Correlation plot of CD69 expression, as analyzed in A, with CD107a expression, as analyzed in figure 3 and 4, of resting and IL-2-treated NK cells. The regression coefficient (r2) and its *P*-value (*P*) were calculated by linear regression analysis.


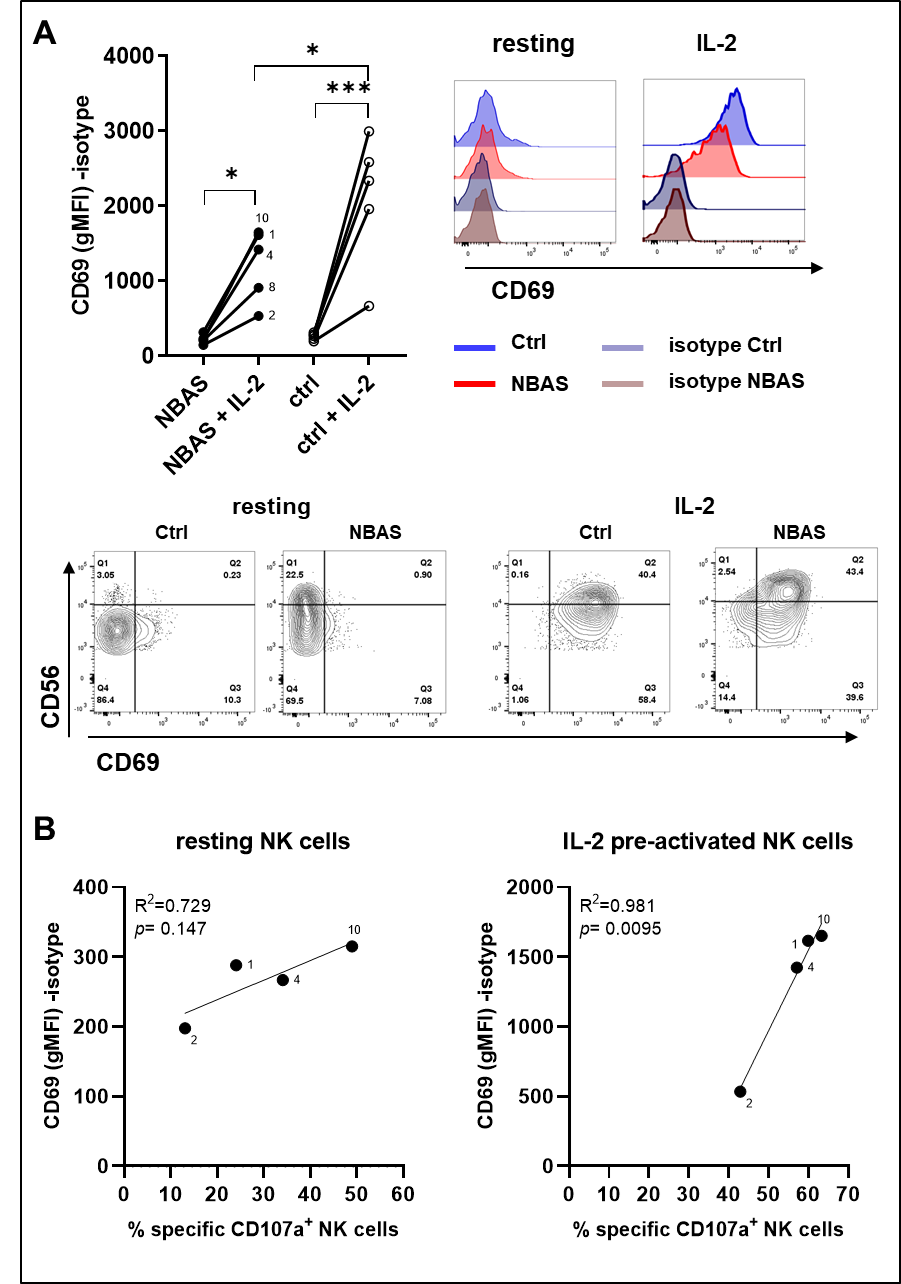

Supplement: Supplementary file 1 — Supplementary file1 (DOCX 359 KB) [file 10875_2021_1110_MOESM1_ESM.docx]
